# Clinical feasibility of the therapeutic strategies total neoadjuvant therapy and “watch and wait” in the treatment of rectal cancer patients with recurrence after clinical complete response

**DOI:** 10.3389/fsurg.2022.1006624

**Published:** 2023-01-16

**Authors:** Dianyin Dai, Ge Liu, Huanran Liu, Yanfeng Liu, Xinlu Liu, Shuang Li, Yanan Lei, Yun Gao, Yuezhu Wang, Shoujia Zhang, Ran Zhang

**Affiliations:** ^1^ Department of Anorectal Surgery, Department of General Surgery, The First Affiliated Hospital of Dalian Medical University, Dalian, China; ^2^ Department of Radiology, The First Affiliated Hospital of Dalian Medical University, Dalian, China

**Keywords:** total neoadjuvant therapy, local recurrence, watch and wait, rectal cancer, circumferential resection margin (CRM)

## Abstract

**Purpose:**

In recent years, total neoadjuvant therapy (TNT) has emerged as a new therapeutic strategy against advanced rectal cancer (RC). After administration of TNT, some patients show complete clinical response (cCR) to treatment however, disputes about the effects of TNT and the alternative treatment plans in case of recurrence after cCR still exist.

**Methods:**

A total of 100 patients were included in this paper. CR and non-CR was observed when these patients were administered with TNT at the First Affiliated Hospital of Dalian Medical University, China from May 2015 to June 2021. These patients received different chemotherapeutic regimens, with close monitoring and watch and wait (W&W) strategy being applied by a multidisciplinary team (MDT). According to treatment results, patients were divided into a cCR group and a non-cCR group; according to the recurrence during W&W, they were divided into a recurrence group and a no-local-recurrence group. This study analyzed the factors that may affect the prognosis, and summarized the surgery and treatment after recurrence.

**Results:**

The TNT strategy was effective, and 85% of patients achieved local remission. However, W&W did not affect the survival time of CR patients, nor did it cause new distant metastasis due to local recurrence during the observation period (*P *> 0.05). However, for patients with positive CRM, we do not recommend W&W as the first choice of treatment (*P *< 0.05).

**Conclusion:**

(1) Whole-course neoadjuvant therapy was an effective treatment scheme for advanced mid-term rectal cancer. The total local reduction rate of this group of cases was 85.00%, meaning that 25 patients achieved CR. (2) W&W was safe and reliable, and CR patients could receive it as the preferred treatment. (3) CRM was an independent risk factor for local recurrence in CR patients. We do not recommend W&W as the preferred treatment for CR patients with positive CRM.

## 1. Introduction

According to GLOBOCAN data from 2020, an estimated 1.9 million new cases of colorectal cancer (CRC) where reported, while about 935,000 deaths were estimated to be attributed to CRC alone (1). Early detection of CRC is often overlooked and most patients are first diagnosed with advanced rectal cancer through colonoscopy during physical examination. Although the survival rate of patients has been significantly improved through the diverse treatment methods available today, improvement in the therapeutic modalities and the post-operation management is pertinent (2–4) since set clinical standards for rectal cancer care are still non-existent.

The European Society of Medical Oncology (ESMO) recommends preoperative neoadjuvant chemoradiotherapy (NCRT) for patients with advanced disease (>T3), with suspected metastatic lymph node involvement, who are medically unsuitable for total mesorectal excision (TME) surgery (5). Most early rectal cancer can be cured by TME (6), due to the uniqueness of the rectal lymphatic drainage system, wherein the close proximity of the rectum to important structures, such as bladder, ureter, prostate of males, and uterus and vagina of females, plays an important part. Therefore, for most patients with advanced rectal cancer and some patients with early rectal cancer the total neoadjuvant therapy (TNT) is the first line of treatment that aims to reduce the extent of the disease to save the surrounding organs and preserve the anus. TNT increases the probability of curative resection (R0) after the highly probable downstaging and reduces both local and distant recurrence (7). TNT has gradually become the mainstream standard treatment strategy and the first line of treatment of choice for patients with advanced rectal cancer, since it reduces the T-grades of the primary tumor and improves the organ preservation rate (OPR), while simultaneously reducing the risk of early micrometastasis among lymph nodes substantially (8). TNT sensitizes the induction and consolidation before and after chemoradiotherapy or neoadjuvant chemotherapy (9).

Since tumors have varied sensitivity to radiotherapy and chemotherapy, 20% of rectal cancer patients show complete clinical response (cCR), while about 10% have pathological complete remission (pCR) after TNT (10). Habr-Gama first proposed the watch and wait (W&W) treatment strategy as the first safe and clinically feasible therapeutic strategy of choice for patients with cCR (11). Thereafter, she published a viable follow-up clinical strategy involving the implementation of stricter physical examination in cCR patients who did not have surgery, which includes: (1) digital rectal examination, rectal endoscopy, biopsy, and blood carcinoembryonic antigen (CEA) level assessments every month in the first year; (2) every 2 months in the second year, (3) every 6 months in the third year in combination with the computed tomography (CT) examination of the thoracoabdominal pelvis every 6 months. Although most patients with cCR benefit from the strict follow-up plan, the low efficacy of the TNT on the tumor, some patients with cCR show local recurrence or distant metastasis during the W&W period (12). Therefore, correctly guiding the patients for follow-up treatments post attainment of the cCR has become a clinical challenge.

## 2. Patients and methods

### 2.1. Source of medical records

The data of this study were taken from 114 patients with rectal cancer who were treated at the First Affiliated Hospital of Dalian Medical University from May 2015 to June 2021 and chose neoadjuvant treatment. Patients who refused surgery were all included in the study.

### 2.2. Inclusion criteria

(1) All cases were evaluated by imaging, physical examination, and various test results, which took place before total neoadjuvant treatment; suspected rectal cancer was diagnosed by digital rectal examination and rectal endoscopy and confirmed by histopathology;
(2) All cases were confirmed to have a T stage >T3 and (or) the highly suspicious metastatic lymph nodes were involved or could not be removed by R0;
(3) Due to invasion of the sphincter, it was impossible to preserve the anus, but the patient had a strong desire to preserve the anus;
(4) Thoracic, abdominal and pelvic imaging examinations were completed before treatment to determine the tumor stage;
(5) All patients received total neoadjuvant treatment before the operation.

### 2.3. Exclusion criteria

(1) Serious life-threatening systemic diseases such as those of the heart, lung, brain, kidney, or liver;
(2) Multicentric or synchronous or recurrent CRC;
(3) Concurrent or previous malignant tumors;
(4) Receipt of preoperative concurrent radiotherapy and chemotherapy only;
(5) Incomplete clinical case data.

## 3. Data collection and sorting

A total of 100 rectal-cancer patients who met the inclusion and exclusion criteria for neoadjuvant treatment were discovered in and collected from our medical-data platform and the Lianzhong medical-record management system.

### 3.1. TNT strategy

All patients were administered TNT, wherein 93 cases were treated with induction chemotherapy (IC)-TNT. Twelve patients were treated with IC-Xeloda® (capecitabine) regimen combined with radiotherapy, while the other 81 cases were treated with IC-Xelox regimen. The remaining patient was treated with simple Xelox (combinatorial chemotherapy involving Xeloda® and oxaliplatin). All patients who were treated with (IC)-TNT were treated with capecitabine combined with radiotherapy after sensitization. Concurrent radiotherapy and chemotherapy regimen include radiotherapy dose of 50 gy in 25 fractions or 2.0 gy per fraction with an oral dosage of 825 mg m^−2^ capecitabine taken twice a day for the duration of radiotherapy, with the initial dose starting approximately 1 to 2 h before radiotherapy. Thereafter, recommendation for dose reductions were applied wherein medications were continued for 5 consecutive days a week, over a period of 5 weeks.

### 3.2. Multidisciplinary-team review

If there were no special circumstances, the patient received enhanced CT or MRI, colonoscopy, and mucosal biopsy 6 weeks after the end of treatment, and the treatment effect was determined after multidisciplinary-team (MDT) review by senior doctors in the anorectal surgery, imaging, radiotherapy, pathology and oncology departments of our hospital. According to the expert consensus on the protection of patients' rights when the W&W strategy is followed after neoadjuvant treatment of rectal cancer (13), CR patients and their families can choose the W&W treatment scheme and provide their consent.

### 3.3. Watch and wait strategy for complete clinical response

After about 1–2 years of obtaining cCR, follow-ups and reexaminations were conducted every 3 months. Follow-ups were conducted every 6 months from the third to fifth year. The patients were followed up once a year from the sixth year. If the patient had additional operative procedures during the follow-up period, the follow-up plan was restarted according to the above rules. Each review included evaluation of the serum CEA, imaging-based examination of the liver, lung, and kidneys, digital rectal examination or colonoscopy, and histopathological examination whenever necessary. Any patients who were diagnosed with suspected local recurrence or definite local recurrence were subjected to another surgery. The specific operation scheme and standards have been enumerated below.

### 3.4 Treatment plan and follow-up plan for patients without clinical response and patients with recurrent clinical response during watch and wait

After the patient and their family members are informed, they decided whether the patient would undergo surgery after consultation.

#### 3.4.1. Patients who refused surgery

Patients who refused surgery were recommended to continue chemotherapy in the oncology department.

#### 3.4.2. Patients who consented to surgery

For patients who agreed to the operation, after chest CT, abdominal CT, enteroscopy, pathology, and other presurgical examinations to ensure there were no distant metastases, the senior doctor of our hospital's First Department of General Surgery performed the operation. Surgical methods were divided into trans-anal local resection, anterior rectal resection with anastomosis (Dixon procedure and ISR procedure) or without anastomosis (Hartmann procedure), or abdominal-perineal amputation (Miles procedure).

### 3.5. Histopathological examination of postoperative specimens

The histological type, degree of differentiation, and depth of invasion of the primary focus were examined. A 0.5 cm section of the severed end of the rectal specimen was cut and evaluated for the presence of tumor residue using microscopic observations of the hematoxylin and eosin (H&E) stained samples. The lymphoid adipose tissue around the root of the inferior mesenteric artery and on both sides of its trunk, the mesorectum, and the lymph nodes in the specimen were studied by microscopic examinations of the histological specimens stained with H&E. Each lymph node had 6 sections for H&E staining and 1 section for cytokeratin immunohistochemical staining, for evaluation of cancer metastasis using microscopic examinations.

### 3.6. Follow-up strategy after surgery

In the first year after surgery, digital examination of the anus was performed every month; CT of head, chest, and abdomen was performed every 3 months; enteroscopy was performed every 3–6 months; and pathological examination was performed when necessary. During the second year, follow-up with an anal re-examination was performed every 3 months, CT and enteroscopy every 6 months, and pathological examination when necessary. At the beginning of the third year, patients were followed up on every 6 months with an anal examination, CT, enteroscopy, and pathological examination when necessary.

### 3.7. Statistical analysis

The SPSS version 26.0 statistical tool was used to analyze the data. The counting data were tested using *t*-test or *Z*-test, and the measurement-based data were tested using the Fisher's test. *P *< 0.05 was considered to be statistically significant. All data were recorded for up to three decimal places.

## 4. Results

### 4.1. Analysis of factors influencing CR after neoadjuvant therapy

#### 4.1.1. Single-factor analysis of clinical data affecting the prognosis of CR

A total of 100 patients were enrolled in this study, of whom 25 received CR after treatment. Among the remaining 75 non-CR patients, 60 were partial response, 14 were stable disease, and 1 was progressive disease. A total of 85 patients (85.00%) successfully achieved local remission, of whom 25 patients received CR.

According to the prognosis evaluation, the patients were divided into a CR group (25, 24.00%) and a non-CR group (75, 75.00%). Thirteen factors that may affect CR were selected for univariate analysis, including gender, age, CEA level before neoadjuvant therapy, pre-T stage, pre-N stage, pre-M stage, distance from the lower edge of the tumor to the anus, neoadjuvant regimen, MRI-circumferential resection margin (CRM), degree of pathological differentiation before neoadjuvant therapy, death, and new distant metastasis. The results showed that none of these 13 factors affected the CR effect in patients with advanced rectal cancer after TNT treatment (Table 1).

**Table 1:** Single-factor analysis of clinical factors affecting prognosis of CR after TNT treatment.

Factor	cCR	No-cCR	*P*
Age			1.000
<65 years	14	40	
>65 years	11	35	
Gender			0.081
Female	4	27	
Male	21	48	
Pre-T stage			0.709
T2	1	1	
T3	19	58	
T4	5	16	
Pre-N stage			0.862
N0	4	12	
N1	17	45	
N2	3	13	
N+	1	5	
Pre-M stage			0.569
M0	25	71	
M1	0	4	
TNT scheme			0.269
IC-Xeloda	3	10	
IC-Xelox	22	58	
Xelox	0	7	
Radiotherapy			0.187
Chemoradiotherapy	25	68	
Chemotherapy	0	7	
CEA			0.635
<5 ng/ml	17	45	
>5 ng/ml	8	30	
Distance from lower edge of tumor to anus			0.387
<5 cm	22	58	
>5 cm	3	17	
Degree of pathological differentiation before neoadjuvant therapy			0.296
High differentiation	4	11	
Medium differentiation	11	48	
Low differentiation	9	16	
CRM			0.628
Negative	49	18	
Positive	26	7	
Death			1.000
Yes	0	2	
No	25	73	
New distant metastasis			1.000
Yes	2	6	
No	23	69	

#### 4.1.2. TNT's impact on survival, prognosis, and choice of follow-up treatment

Of the 100 patients in this group, 2 died of their tumors and 98 survived. Follow-up time was 9–87 months (average, 34.14 months). The overall-survival (OS) rate was 98.00% and the cancer-specific survival (CSS): 98.00%. Of all non-CR patients, a total of 66 underwent surgery within 6–8 weeks after total neoadjuvant therapy, and the remaining 9 patients refused surgery due to intolerability and personal reasons.

**Figure 1:**
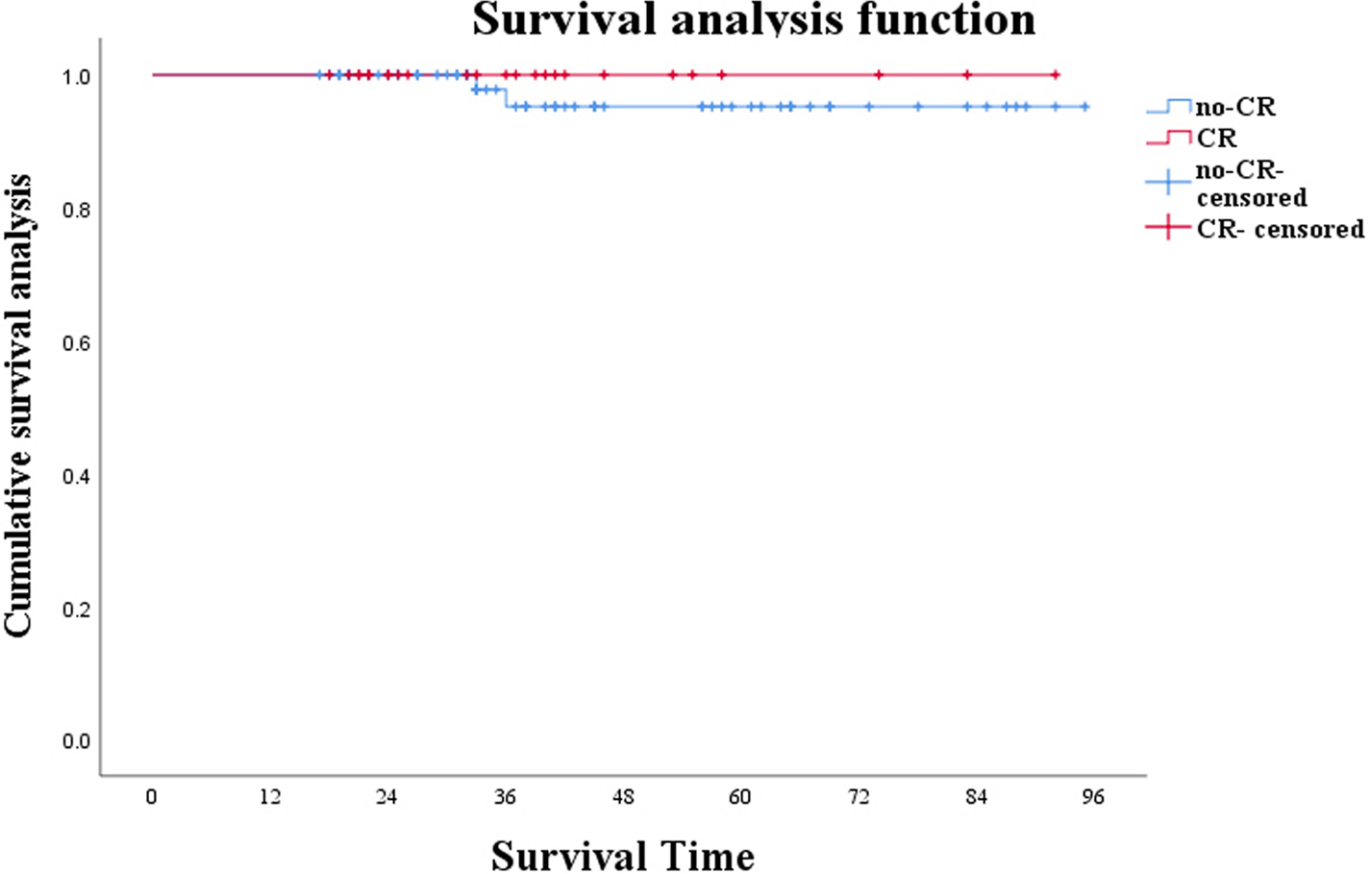
Survival analysis of patients under W&W follow-up strategy and those showing no cCR (*P *= 0.419).

**Figure 2:**
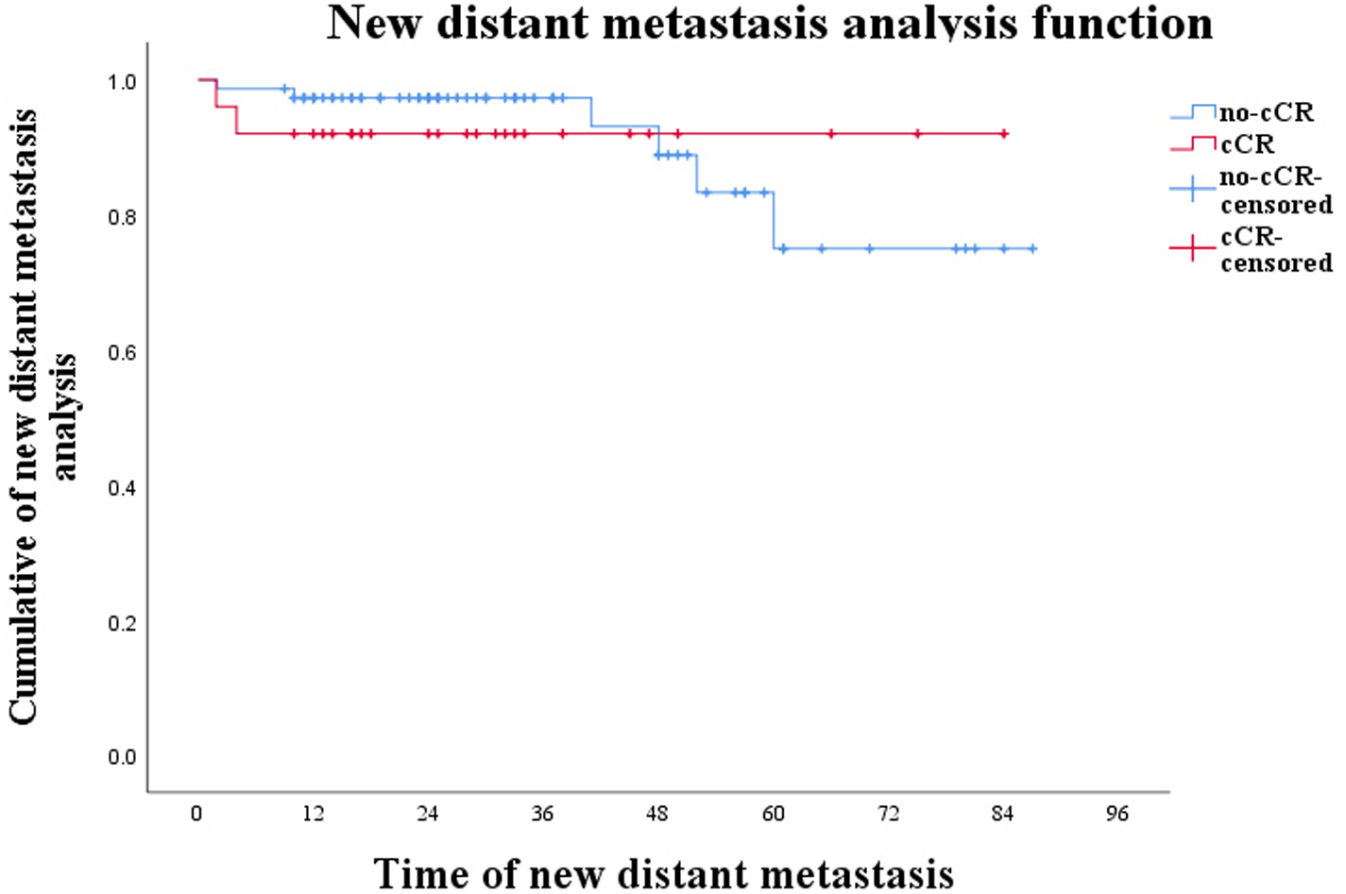
Curve of new distant metastases in CR and non-CR patients (*P *= 0.806).

Preoperative evaluation included enhanced CT or MRI, colonoscopy, and mucosal biopsy, and findings were used to select surgical procedures. These included 3 cases of ycT1N0M0 and negative CRM who accepted LE, 17 cases of Dixon, 22 of Miles, 22 of ISR, and 2 of Hartman (Table 2). The operation rate was 66.00%, the sphincter preservation rate (SPR) was 63.64%, and the organ preservation rate (OPR) was 4.55%. Combined with the survival curves of the two groups of patients, W&W did not shorten the survival time of CR patients (Figure 1; *P* = 0.419), nor did it increase the risk of new distant metastases (Figure 2; *P* = 0.813).

**Table 2:** Follow-up treatment plan for non-CR patients.

Operation	No. of cases	Percentage	Cases of mortality	New distant-metastasis cases
LE	3	4.54%	0	1
Dixon	17	25.76%	0	0
ISR	22	33.33%	0	5
Miles	22	33.33%	0	0
Hartman	2	3.03%	1	0

### 4.2. Analysis of factors affecting local recurrence in CR patients

#### 4.2.1. Univariate analysis of local recurrence in CR patients

After preoperative neoadjuvant therapy, a total of 25 patients successfully obtained CR status through TNT protocol and then were closely followed up on. During this period, five patients were diagnosed with local recurrence. According to the recurrence during the follow-up period, we divided patients into a recurrence group (*n* = 5, 20.00%) and a no-local-recurrence group (*n* = 20, 80.00%). We selected 11 factors that may affect recurrence for analysis, including gender, age, pre-T stage, pre-N stage, neoadjuvant regimen, CEA level before treatment, degree of pathological differentiation before neoadjuvant, distance from anus, CRM, and new distant metastases. The results showed that CRM was the factor affecting recurrence after CR (*P* < 0.05; Table 3).

**Table 3:** Single-factor analysis of clinical factors affecting prognosis of local recurrence during W&W. CRM had a significant effect on local recurrence in cCR patients during W&W.

Factor	No-local-recurrence	Local recurrence	*P*
Age			0.341
>65 years	10	4	
<65 years	10	1	
Gender			0.549
Female	4	0	
Male	16	5	
Pre-T stage			1.000
T2	1	0	
T3	16	5	
T4	3	0	
Pre-N stage			0.667
N0	4	0	
N1	13	4	
N2	2	1	
N+	1	0	
TNT scheme			1.000
IC-Xeloda	3	0	
IC-Xelox	17	5	
CEA			0.283
<5 ng/ml	15	2	
>5 ng/ml	5	3	
Degree of pathological differentiation before neoadjuvant therapy			0.670
High differentiation	6	1	
Medium differentiation	10	2	
Low differentiation	4	2	
Distance from lower edge of tumor to anus			0.091
Less than 5 cm	19	3	
Greater than 5 cm	1	2	
CRM			0.023
Negative	16	1	
Positive	4	4	
New distant metastasis			1.000
Yes	2	0	
No	18	5	

#### 4.2.2. Multifactor analysis of CR patients' recurrence

Univariate-analysis *P* < 0.1 factors affecting local recurrence of CR patients, including CRM and the distance between the lower edge of the tumor and the anus, were included in the binary logistic regression. The results showed that CRM (0.049, 0.074, 0.006–0.992) was an independent risk factor affecting recurrence in CR patients (Table 4).

**Table 4:** Multivariate analysis of clinical factors affecting local recurrence during W&W. CRM (0.049, 0.074, 0.006–0.992). CRM was an independent risk factor for local recurrence during W&W, while distance from the lower edge of the tumor to the anus (0.108, 0.108, 0.004–2.670) had no significant effect.

Factor	B	Standard Error	Wald	df	*P*	Exp (*B*)	95%CI	
							Lower limit	Upper limit
CRM	−2.604	1.324	3.866	1	0.049	0.074	0.006	0.992
Distance from lower edge of tumor to anus	−2.222	1.635	1.847	1	0.174	0.108	0.004	2.670

#### 4.2.3. Treatment of suspected recurrent patients under the watch & wait strategy

During the follow-up period, the OS rate and CSS of all CR patients were 100.00%. A total of 12 patients had suspected local recurrence and distant metastases, including 10 with suspected recurrence and 2 with distant metastasis. One of the 10 patients with suspected recurrence refused surgery due to the low location of the tumor. The remaining nine were treated with remedial surgery according to doctor's advice. Preoperative evaluation, including digital examination of the anus, enhanced CT or MRI, colonoscopy, and mucosal biopsy, and findings were used to select surgical procedures. These included 3 cases of ycT1N0M0 who was accepted LE, one case of Dixon, two cases of ISR, and three cases of Miles. SPR was 88.00% and OPR was 88.00%. Four of the nine patients were diagnosed as having local recurrence by postoperative pathology, and the remaining five obtained pCR status. The recurrence rate was 20.00%, the new distant-metastasis rate was 8.00% and the pCR rate was 20.00%. All patients with definite local recurrence achieved R0 resection; except for one who had a recurrence at the 26th month, the rest had recurrences within 24 months. Compared with before neoadjuvant therapy, their T-stage decreased to varying degrees, and no positive lymph nodes were detected. During the follow-up period, a total of two patients developed local CR with distant metastases, including one with bone metastasis during the 8th week after the end of treatment and one with liver metastasis at the 7th week after the end of treatment. These two patients chose to continue chemotherapy (Tables 5, 6).

**Table 5:** Clinical data of 11 patients with suspected local recurrence who were accepted for remedial surgery but diagnosed with distant metastasis.

Patient no.	Gender	Age	Distance from tumor to anus before TNT	Initial stage	Operation interval/distant-metastasis time (months)	Type of remedial surgery	Postoperative T-stage	No. of detected lymph nodes vs. positive number	Stoma condition and type	Survival time (months)	Survival state
9	Male	58	3–8	T3N0	12	APR	pCR	5–0	Permanent colostomy	69	No tumor
30	Male	60	4	T4N1	8	LAR	pCR	6–0	—	47	No tumor
31	Male	45	2–5	T4N1	32	LE	pCR	—	—	39	No tumor
40	Male	61	2–7	T3N1	26	APR	4	8–5	Permanent colostomy	33	No tumor
44	Male	61	2–6	T3N1	8	LE	pCR	—	—	26	No tumor
45	Male	58	6–11	T3N2	16	LAR	2	12–0	—	24	No tumor
50	Male	62	5–10	T3N1	12	LAR	3	7–0	—	23	No tumor
51	Male	55	5–10	T3N1	26	LE	pCR	—	—	28	No tumor
69	Male	62	5	T3N1	12	APR	3	2–0	Permanent colostomy	18	No tumor
79	Male	70	4	T3N1	<2					14	Local cCR with bone metastasis
80	Female	81	4	T3N0	<2					13	Local cCR with liver metastasis
82	Male	77	3-8	T3N1	2	Declined surgery	—	—	—	11	Tumor-bearing

**Table 6:** Comparison of descending phases of T-stage patients who were suspected to have local recurrence and accepted for remedial surgery after administration of W&W.

**T-and N-staging of tumor before TNT treatment**	
**cT**	
3	7
4	2
**Postoperative pathology**	
**ypT**	
0	5
1	0
2	1
3	2
4	1
**Descending period — T**	
−4	2
−3	3
−2	0
−1	1
0	2
+1	1

**Figure 3:**
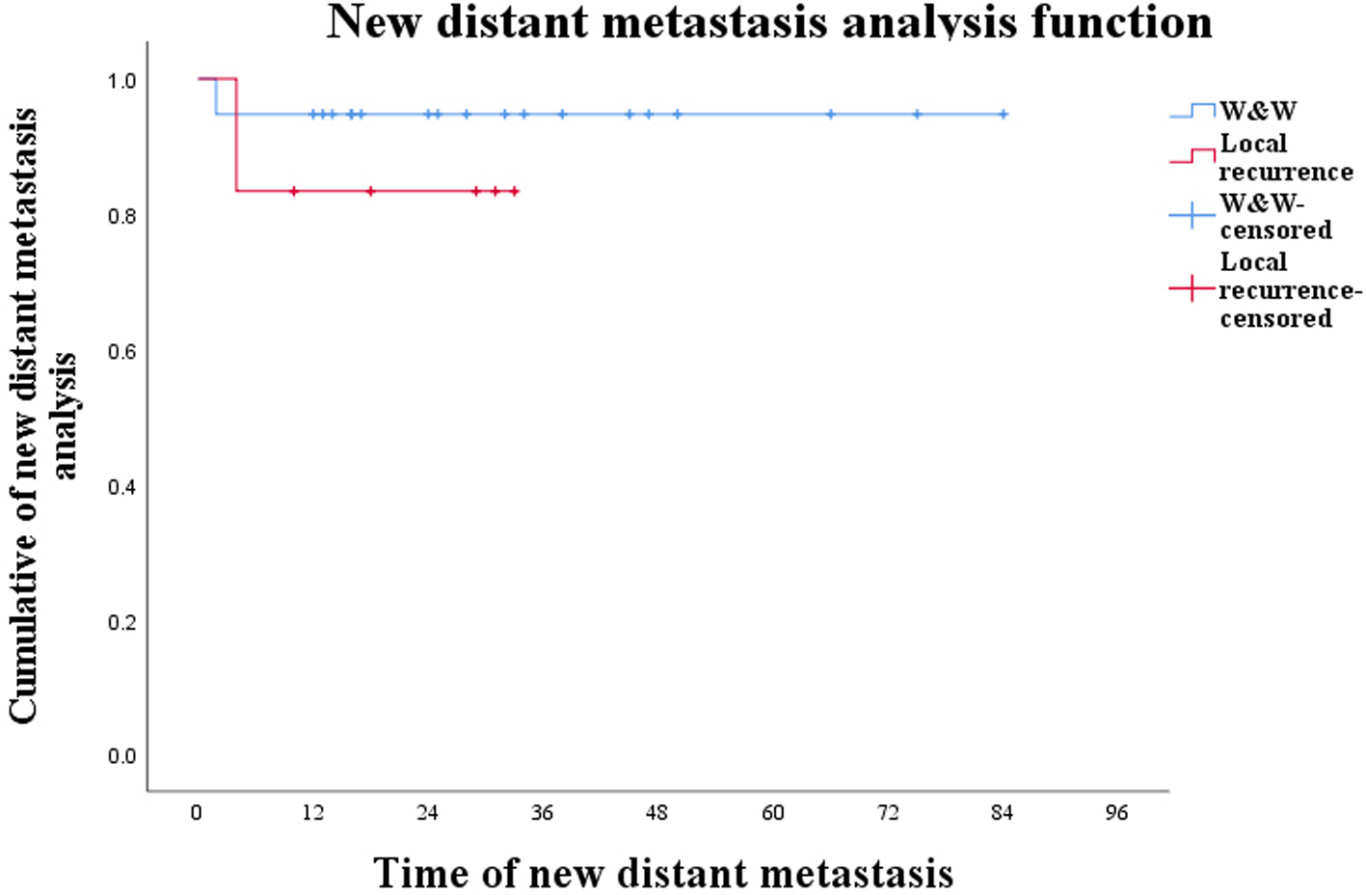
Curve of new distant metastases in W&W patients and local-recurrence patients during W&W (*P *= 0.474).

In conclusion, although W&W posed the risk of local recurrence and new distant metastases, in this study they did not affect patients' survival time while local recurrence did not affect survival time in patients with new distant metastases (Figure 3; *P* = 0.474).

## 5. Discussion

Consorted studies carried out with patients having rectal cancer are continuously transforming the guidelines and principles for the treatment of advanced rectal cancer. Although at present TME is the preferred therapeutic modality of choice in most patients with rectal cancer, instances of local recurrence, distant metastasis, permanent colostomy, and impact of colostomy on patients' psychology and physiology are compelling clinicians to explore more clinically feasible therapeutic options for the treatment of advanced CRCs (8). Combining TNT with TME surgery can reduce the recurrence rate and improve the SPR and OPR. The core idea of NCRT is to reduce malignancy through TNT to reach the clinically feasible therapeutic goal of curative surgery or W&W (14). TNT aims to increase the proportion of pCR patients based on NCRT (13).

A total of 115 patients at our center chose neoadjuvant therapy, of whom 15 were excluded because they did not meet the conditions for inclusion. Although this study was a single-center study, most patients (85.00%) successfully benefited from neoadjuvant therapy. First, we divided neoadjuvant-therapy patients into a CR group and a non-CR group to explore the impact of various factors on prognosis after neoadjuvant therapy. The results showed that 13 factors such as gender were not independent risk factors after TNT, but gender had a greater impact on the prognosis of CR (*P* = 0.063; 0.328, 0.101–1.063). Some researchers believe that degree of pathological differentiation is an independent risk factor after TNT (15) and directly affects patient survival time (16), but we did not reach a similar conclusion after comparing the three degrees of pathological differentiation between the CR and non-CR groups. In addition to degree of differentiation, clinical T-stage before treatment is also an important factor affecting tumor response to treatment and indirectly affects the survival time of patients (17). It might be that single-center research and too few samples indirectly weakened the influence of the above factors on the TNT effect. All patients chose the treatment plan after MDT evaluation after TNT treatment. Except for one patient who underwent surgery in the second week after the end of treatment due to intestinal obstruction, all patients were evaluated at the sixth week after neoadjuvant treatment. Observation time after neoadjuvant treatment should be 6–12 weeks, which is in line with most international guidelines at present. Due to the delayed retraction of tumors, some near-CCR patients enter CCR 13–49 weeks after treatment. Therefore, some researchers believe that the observation time window should be appropriately widened on the basis of strict follow-up and reexamination (13), and it is suggested that suspected CR patients undergo more-rigorous secondary evaluation (18, 19). We will re-plan the evaluation time in the future and strive to re-evaluate patients with near CCR on the premise of ensuring safety so as to maximize OPR and SPR.

Of the 75 non-CR patients screened by the MDT after treatment, 9 refused surgery due to physical reasons, and the remaining 66 received surgical treatment. Except for 3 cases of Le and 2 cases of Hartman, the remaining 61 patients achieved R0 resection. By comparing the survival time and new metastases of the two groups and analyzing their long-term prognoses, we concluded that W&W was safe and reliable (*P* = 0.374) and did not cause new distant metastases (*P* = 0.806). Since Habr-Gama first proposed the W&W strategy in 2004, most studies (20–22) in recent years have reached similar conclusions: this treatment strategy is safe and effective and can be the first choice of treatment for patients with CR, but the feasibility and safety of the W&W scheme must be based on strict diagnostic criteria. Habr-Gama believes that the feasibility of the “observation and waiting” method depends on the accurate identification of patients with cCR by strict standards. Endoscopy, pathological examination, and digital anal examination must be combined (23): digital rectal examination cannot touch any irregular tumors; the surface of the rectal wall must be regular and smooth; and there must be no residual mass, ulcer, or stenosis under the endoscope except for white mucosal scars or telangiectasia (24). However, because there may be residual tumor cells in the deep layer of the rectal wall, and the distribution of residual tumor cells in the intestinal wall has nothing to do with clinical or pathological lymph node status (25), some studies emphasize that local mucosal biopsy is not an absolutely safe examination method and that a full-thickness local excision (FTLE) should be performed when necessary. However, FTLE produces scarring and causes severe pain in low-segment patients and is therefore not conducive to follow-up and anal preservation (24, 26). Therefore, to improve the probability of anal-sphincter preservation after recurrence, we did not rely on FTLE during follow-up in this study.

Although TNT can inhibit and kill the primary tumor and early lymph node micrometastasis to a certain extent so that some patients can obtain CR and avoid surgery *via* the W&W protocol, and although W&W is safe and reliable, this protocol still poses the risk of recurrence. In our analysis of factors affecting the recurrence of 25 CR patients during W&W, the recurrence and new distant-metastasis rates of CR patients were 25.00% and 8.00%, respectively. We divided the patients who attained CR and selected W&W into a recurrence group and a no-local-recurrence group. Ten factors that may affect recurrence, such as gender, were analyzed. The results showed that positive CRM was an independent risk factor for recurrence during W&W. Because the outermost boundary of the mesentery is defined by the mesenteric fascia (MRF), and MRF is used as the barrier for tumor diffusion, CRM positivity is defined as the boundary between the deepest tumor invasion and MRF being <1 mm. This study used this standard for CRM positivity. Some researchers believe that positive CRM is a risk factor for local recurrence and that the distance between the tumor and MRF is negatively correlated with the prognosis (12, 27). A study on neoadjuvant therapy shows that even if patients with positive CRM undergo surgical resection of the lesion after neoadjuvant therapy, positive CRM is still a risk factor for local recurrence (28). We speculate that because the tumor cells are deeply embedded in the intestinal wall, the killing effect of chemotherapy and radiotherapy on the tumor is weakened, so that some live tumor cells enter a dormant state. With an increase in observation time, dormant tumor cells resume proliferation, causing local recurrence. Although we usually use MRI or enhanced CT to evaluate preoperative CRM, ignoring the difference between MRI-CRM and pathological CRM (P-CRM), a prospective study has proven that MRI has >90% sensitivity in differentiating P-CRM (29). In addition to CRM, some studies believe that insufficient differentiation can significantly increase the invasiveness of tumors and the probability of recurrence (30, 31). Of the four cases of poorly differentiated cancer in the CR group, two (50%) had local recurrence. The CRM of patient no. 40 (one of these two patients) was positive, and pathology results indicated low-adhesion cancer. Therefore, we recommended that he be closely followed up every month after attaining CR. Although his compliance was high, he was suspected of recurrence at the 26th month and underwent remedial surgery [abdominoperineal resection (APR)]. The postoperative pathological stage was ypt4bn2m0. Compared with before treatment, the patient's T-stage and N-stages were increased. It might be that CRM-positive patients with poorly differentiation cannot attain CR after TNT treatment, leading to the lack of universality of this conclusion. In addition to the abovementioned two factors, Renehan AG (32) analyzed the recurrence factors of W&W and follow-up treatment after neoadjuvant therapy. The results showed that T-stage, N-stage, and CEA levels before neoadjuvant therapy affected the follow-up treatment plan. Patients with lower levels of these factors could undergo W&W as the preferred treatment strategy, but smoking history and male gender were high-risk factors for recurrence during W&W. We speculate that there were fewer CR patients in our center due to poor regression of some tumors with high T-stage or N-stage or high CEA and insensitivity of poorly differentiated tumors to neoadjuvant therapy, which weakens the influence of pathological differentiation, CEA level, and T stage on recurrence. Although gender had no statistically significant effect on local recurrence, patients with recurrence were all male and accounted for 30% of the total. Because this was a retrospective study, there was a bias in the accuracy of recurrence time in some patients with low compliance.

In addition to local recurrence, new distant metastasis is another important factor affecting the survival of CR patients. Unfortunately, 2 patients attained local CR after neoadjuvant therapy but one developed liver metastasis at week 7 and the other developed thigh-bone metastasis at week 8. Although TNT has added a sensitization or consolidation scheme to its original basis for the purposes of inhibiting early micrometastasis and enabling local radiation to kill tumor cells in order to shrink the tumor body, this method still mainly depends on the killing effect of radiation on the primary tumor, and its effect on early distant micrometastases is still not ideal. Although most patients who choose TME after radiotherapy and chemotherapy can achieve R0 resection, they still have distant metastases, which greatly reduces the long-term survival rate (26, 33). This is also the main reason some advanced rectal-cancer patients with new distant metastases after TNT treatment cannot benefit from TNT. In the case of local CR with distant metastasis, we speculate that before neoadjuvant therapy, a small number of circulating tumor cells (CTCs) have colonized the surfaces of organs (34) and formed early micrometastases. Therefore, how to accurately locate and inhibit CTCs in the early stage and enhance the inhibitory effect of TNT on early micrometastasis has become our next goal.

Since the pelvic anatomy of W&W patients was not involved in surgery, the success rate of remedial TME surgery was high. The SPR and OPR of this group of patients were 88.00% and 76.00%, respectively, and all patients achieved R0 resection. Postoperative pathological results also proved that TNT had a significant inhibitory effect on depth of invasion and tumor–lymph node metastasis after recurrence of the primary focus, achieving the expected effect (8). Wang (35), using oxaliplatin plus capecitabine (CC-CapOX) in patients with advanced middle rectal cancer, not only obtained a high level of cCR (42.1%) and a very low metastasis rate (7.6%) but was able to preserve the anus in >90% of patients, which was similar to our results.

Because radiotherapy increases the probability of pelvic and small-intestinal injury, the TNT regimen has opened up a new treatment idea of chemotherapy alone, reducing the side effects and injury caused by radiotherapy and surgery. However, some researchers believe that TNT's pCR rate is lower than that of radiotherapy (19). Pathological CR is the ultimate goal of neoadjuvant therapy. Habr-Gama et al*.* first proposed that consolidation chemotherapy in the waiting period can also increase the pCR rate. They registered 70 patients with rectal cancer within 7 cm of the anus in cT2-T4 or cN1-N2. After patients completed neoadjuvant radiotherapy and chemotherapy, they increased the same chemotherapy cycle to once a week (>6 weeks in total) and evaluated the tumor twice *via* endoscopy combined with MRI, 6 and 10 weeks after treatment. The results showed that after an average follow-up of 53 months, 39 patients (51%) achieved continuous cCR, with 3-year OS and disease-free survival (DFS) of 94% and 75%, respectively (36, 37). Surgical interval is another important factor affecting pCR. Tulchinsky (38) et al*.* found that the pCR rate of patients with a surgical interval of >7 weeks after neoadjuvant therapy is 35%, while that of patients with a surgical interval of <7 weeks (17%) is less than half that of the former group of patients (*P* = 0.03). Zeng et al*.* (39) reached the same conclusion, that the pCR rate of patients with a surgical interval of >7 weeks is higher (27.1% vs. 15.3%). During the follow-up period of this study, a total of nine patients with suspected recurrence chose surgery. The interval between operations ranged from 8 to 26 months, averaging 16.89 months. Postoperative pathological results showed that the pCR rate was 20.00%, which was similar to the results of above-mentioned study.

TNT gives patients with low rectal cancer a better chance of preserving the anus. However, due to postsurgical changes to the rectum and morphology, most patients have abnormal defecation, changes in stool characteristics, and bowel control disorders (40), which seriously affects their quality of life. Some researchers believe that low rectal cancer, radiotherapy, and pelvic autonomic nerve injury are risk factors for such intestinal dysfunction (18, 41). Therefore, to balance the effect of neoadjuvant therapy with preservation of the anus, postoperative intestinal dysfunction should be predicted, and corresponding preventive or therapeutic measures should be taken.

This study had certain limitations. First, the current CR standard cannot accurately predict PCR. We should collect additional patient- and tumor-specific information as a basis for building a more stable and accurate model. Second, from the perspective of patient safety, the center adds radiotherapy to the treatment plan of most patients with advanced rectal cancer but ignores the advantages of chemotherapy alone. Therefore, in the future we will conduct a prospective study, add immunotherapy on the premise of concurrent radiotherapy and chemotherapy or chemotherapy alone, and compare the short- and long-term effects of the various strategies.

To sum up, most patients with advanced rectal cancer could benefit from neoadjuvant therapy. Some patients would attain CR, reducing the physiological or psychological pressure that the W&W strategy imposes on them. At the same time, the TNT regimen could not only preserve the anus or organs of patients with advanced rectal cancer but also effectively inhibit early lymph node metastasis and reduce the depth of tumor invasion after recurrence.

## 6. Conclusion

(1) Whole-course neoadjuvant therapy was an effective treatment scheme for advanced mid-term rectal cancer. The total reduction rate of this group of cases was 85.00%, meaning that 25 patients attained CR.
(2) W&W was safe and reliable, and CR patients could undergo it as the preferred treatment.
(3) CRM was an independent risk factor for local recurrence in CR patients. We do not recommend W&W as the preferred treatment for CR patients with positive CRM.

## Data Availability

The raw data supporting the conclusions of this article will be made available by the authors, without undue reservation.
